# The Potential of Crude and Partially Purified Black Rice Bran Extracts Obtained by Ultrasound-Assisted Extraction: Anti-Glycemic, Cytotoxicity, Cytoprotective, and Antitumoral Effects

**DOI:** 10.3390/foods13040597

**Published:** 2024-02-16

**Authors:** Eduardo Leonarski, Mayara Kuasnei, Eloisa H. Santos, Paulo A. D. Moraes, Karina Cesca, Débora de Oliveira, Acácio A. F. Zielinski

**Affiliations:** 1 Department of Chemical Engineering and Food Engineering, Federal University of Santa Catarina (UFSC), Florianópolis 88010-970, SC, Brazil; eduardoleonarski@gmail.com (E.L.); mayara_kuasnei@hotmail.com (M.K.); ds.eloisa@gmail.com (E.H.S.); karinacesca@gmail.com (K.C.); debora.oliveira@ufsc.br (D.d.O.); 2 Department of Chemistry, Federal University of Santa Catarina (UFSC), Florianópolis 88010-970, SC, Brazil; paulo.a.d.moraes@ufsc.br

**Keywords:** *Oryza sativa* L., purification, oxidative stress, antidiabetic potential

## Abstract

Recovering anthocyanins from black rice bran is a way of valuing this byproduct, by obtaining an extract with biological potential. The objective of this study was to recover anthocyanins using ultrasound-assisted extraction. Some of the extract was partially purified, and both (crude and partially purified) extracts were evaluated for their anthocyanin content, antioxidant activity, antidiabetic and antitumoral activities, cytotoxicity, and oxidative stress. An increase in the laboratory scale was also achieved, making possible to increase the extraction volume up to 20 times without significantly changing the content of anthocyanins (1.85 mg C3G/g DW). It was found that the purified sample presented a 4.2 times higher value of total anthocyanins compared to the crude sample. The best IC_50_ values for the purified sample were verified by DPPH and ABTS (0.76 and 0.33 mg/mL). The best results for antidiabetic activity were obtained for the partially purified sample: 0.82 µM C3G for α-glucosidase and 12.5 µM C3G for α-amylase. The extracts demonstrated protection (~70%) when subjected to the oxidative stress of L929 cells. An antitumoral effect of 25–30% for both extracts was found in A459 cells. The crude and partially purified extracts of black rice have antidiabetic and anticancer effects and more studies are needed to explore their potential.

## 1. Introduction

Black rice processing produces about 10% rice bran, 14% broken rice, and 20% rice husk [1,2,3], which also has high functional value mainly due to its high anthocyanin content [4,5]. Black rice bran is a rich anthocyanin source, which has been reported to have antidiabetic effects by inhibiting the activities of α-amylase and α-glucosidase (reducing the risk of type 2 diabetes) [6,7]. Furthermore, it was also found that black rice bran extract has a cytoprotective effect on H_2_O_2_-induced oxidative stress in L292 cells, indicating that these extracts may have protective effects against oxidative reactions [5]. Therefore, the recovery of these extracts which are rich in anthocyanins may be interesting for evaluating their biological potential.

One way to recover these bioactive compounds is through extraction, using, preferably, eco-friendly methods and solvents [8]. The ultrasound method produces waves that achieve a greater penetration into the cellular material, requiring less time and less solvents, in addition to using less energy and allowing for the extraction of heat-labile compounds [9,10]. The expansion of the laboratory scale for UAE is important for the recovery of bioactive compounds, although few studies have been carried out with this objective [11]. This is because scaling up is not a trivial task that only increases the amount of biomass and solvent required; a more in-depth study is necessary, and, therefore, it is necessary to carry out tests from the laboratory scale (cells from 10 to 1000 mL) to the pilot scale (10 to 50 L), and up to industrial scale [9]. The extracts obtained during the process, in addition to being rich in anthocyanins, contain several other compounds, which are called crude extracts.

Crude anthocyanin extracts from black rice contain several other compounds (mainly sugars), which may affect their biological effects and possible applications. Therefore, the use of a macroporous resin (e.g., XAD-7HP, AB-8, HP-20, D-101, or X-5) is a good alternative to partially purify the anthocyanins from plant materials [12]. Through hydrophobic bonding and aromatic stacking, these synthetic resins adsorb to the phytochemicals from aqueous solutions and desorb in organic solvents such as methanol and ethanol [13]. The partial purification of phenolic extracts could improve their antioxidant activity and present an improvement in their anti-inflammatory effect, making them a more effective alternative for treating diseases [14]. 

Anthocyanin extraction from black rice bran by ultrasound and partial purification with Amberlite XAD-7HP resin could be an alternative method to obtain an anthocyanin-rich extract with high biological potential. Therefore, this study aims to recover anthocyanins from black rice bran using ultrasound-assisted extraction (UAE) and purify them using Amberlite XAD-7HP resin. Then, the crude and partially purified extracts’ antioxidant activity, antidiabetic potential, cytotoxicity, and oxidative stress will be evaluated.

## 2. Materials and Methods

### 2.1. Sample Preparation

Black rice bran (BRB) was supplied by Ruzene (Pindamonhangaba, Brazil) and prepared according Leonarski et al. [5].

### 2.2. Ultrasound-Assisted Extraction (UAE)

At the lab scale, the extraction was carried out using an ultrasonic probe (Eco-Sonics, Ultronique Q3.0/37A, Indaiatuba, Brazil), a mass of 0.5 g of BRB, and a 15 mL volume of solvent. The following conditions parameters were used: a temperature of 50 °C, frequency power of 380 W, and a solvent ratio to 60% citric acid (0.1 mol/L) to 40% ethanol. 

For the scale-up, with the same ultrasonic probe used at the lab scale, an experiment was carried out to increase the extraction volume from 15 to up to 300 mL (an increase of 20 times). For this, firstly, the real ultrasound power (P) was measured, considering that the real input power is converted into heat dissipated in the medium, which is determined by calorimetry, as in Equation (1), according to Carail et al. [15]:

(1) P=m·Cp·(dT/dt)

where Cp is the heat capacity of the solvent at a constant pressure (J/g K), m is the mass of the solvent (g), and dT/dt is the temperature rise per second.

Using the power calculated in Equation (1), the consequent ultrasonic intensity (UI) was calculated for the ultrasonic probe, as shown in Equation (2).

(2) $$UI=4P/\pi D^{2}$$

where UI is the ultrasonic intensity (W/cm^2^), P is the ultrasound power (W) as calculated by Equation (1), and D is the internal diameter (cm) of the probe tip.

By calculating the real power (P) and the ultrasonic intensity (UI), it was possible to verify the required power increase when increasing the reactor size to carry out the scale-up. The instrumental parameters are presented in Table 1.

### 2.3. Total Monomeric Anthocyanin (TMA)

The total monomeric anthocyanin (TMA) analysis was performed according to the differential pH methodology [16], as described by Leonarski et al. [5]. The TMA concentrations were calculated using Equations (3) and (4):

(3) $$A_{.}=(A_{520}-A_{700}{)}_{pH\;1.0}-(A_{520}-A_{700}{)}_{pH\;4.5}$$

(4) $$TMA=(A_{.}\cdot MW\cdot DF\cdot 1000)/\varepsilon$$

where $A_{.}$ = absorbance, MW = molecular weight (cyanidin-3-glucoside: 449.2 g/mol), DF = dilution factor (15), and ε = the molar absorptivity of cyanidin (26,900).

#### 2.3.1. Partial Purification of Anthocyanin-Rich Extracts

**Figure 1:**
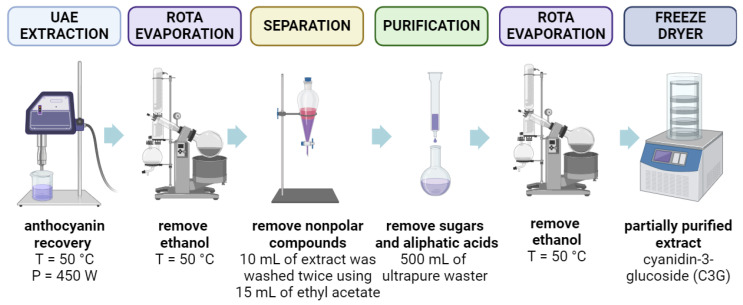
Extraction, partial purification, and freeze-drying steps to obtaining anthocyanins from BRB. Figure created with BioRender.com.

The steps for the extraction of anthocyanins, from black rice bran to freeze-drying, are shown in Figure 1. The anthocyanin-rich extracts obtained using the UAE technique were concentrated, eliminating all ethanol through vacuum rotary evaporation (Fisatom, model 801, São Paulo, SP, Brazil). Using 15 mL of ethyl acetate, 10 mL of concentrated extract was washed twice. A glass column (1.0 cm × 30 cm) was filled with 50 g of Amberlite XAD7HP resin (Sigma-Aldrich, Steinheim, Germany), and the extract was added. Elution was carried out with ultrapure water to remove sugars and aliphatic acids, using a peristaltic pump (Watson-Marlow 323 Series Drive, Falmouth, UK) with a flow rate of 6 mL/min, until reaching a final volume of 500 mL. With a hydroethanolic acid solution [containing 50% (*v*/*v*) ethanol and 1% of acetic acid or citric acid (*v*/*v*)], the purified extract was desorbed by elution. By rotary evaporation, the recovered solution was concentrated and lyophilized.

#### 2.3.2. Determining the Individual Anthocyanins by HPLC-MS

The individual anthocyanins were determined by HPLC-MS and quantified by HPLC-PDA, as described by Leonarski et al. [5]. The lyophilized extracts were resuspended in formic acid 0.1% and filtered through a 0.22 µm filter. The samples (10 µL) were injected into a high-performance liquid chromatography system (model LCMS-2020, Shimadzu, Kyoto, Japan). The equipment consists of a photo-diode array detector (PDA), an LC-20AD binary pump, a SIL-20AC HT autosampler, a central controller, and a single-quadrupole MS detector (Shimadzu) with electrospray ionization (ESI). A Kromasil^®^ C18 column (100 Å, 300 mm × 4.6 mm i.d.) was used. Cyanidin-3-glucoside (C3G) was quantified in mg/g.

### 2.4. Antioxidant Activity

The extracts’ antioxidant potential was evaluated by DPPH and ABTS radical scavenging methods according to Brand-Williams et al. [17] and Re et al. [18], respectively, with adaptations to the microplate reader. The results were expressed as IC50 values (mg/mL).

### 2.5. Effects of Crude and Partially Purified Anthocyanin Extracts on α-Amylase and α-Glucosidase Inhibition

For the α-glucosidase assay, UAE extracts were diluted in phosphate buffer (0.1 mol/L, pH 6.9) and incubated at 25 °C for 10 min with 100 µL of α-glucosidase solution (0.5 U/mL). Then, 50 µL of pNPG (5 mmol/L in phosphate buffer) was added and the solution was incubated for 5 min at 25 °C. By adding 80 µL of sodium carbonate (0.2 mol/L), the reactions were stopped, and, finally, the absorbance was measured at 405 nm [19]. For the α-amylase assay, 40 µL of the extracts, diluted in buffer (0.1 M with 0.006 M NaCl, pH 6.9), was mixed with 150 µL of water, 400 µL of starch solution (0.5%), and 200 µL of enzyme solution (0.5 mg/mL), prepared in phosphate buffer (pH 6.8), and incubated at 25 °C for 15 min. The reaction was stopped using 400 µL of DNS reagent (3.5-dinitro salicyl) and the solution was maintained at 90 °C for 15 min, before, finally, the absorbances were read at 540 nm using a spectrophotometer [20]. The % inhibition of both assays was calculated according to Equation (5):

(5) $$\%inhibition=\left[1-(C-D)/\left(A-B\right)\right]\cdot 100$$

where A = control (with enzyme and without sample), B = control blank (no enzyme and no sample), C = reaction (with enzyme and sample), D = reaction blank (without enzyme and with sample).

### 2.6. Cell Culture

#### 2.6.1. Culture Conditioning 

In Dulbecco’s Modified Eagle Medium (DMEM), cell lines were cultured and supplemented with 10% fetal bovine serum (FBS) and 1% penicillin and streptomycin. The cells were maintained in a humidified environment at 37 °C with 5% CO_2_. The cell culture medium was renewed every 48 h until the cells attained a confluence level of 75–90%. Subsequently, using Tryplex, the cells were dissociated, counted, and seeded.

#### 2.6.2. Cytotoxicity Assay

Using a colorimetric method with MTS [3-(4,5-dimethylthiazol-2-yl)-5-(3-carboxymethoxyphenyl)-2-(4-sulfophenyl)-2H-tetrazolium colorimetric assay], the cytotoxicity of the extracts was evaluated by measuring the metabolic activity of a normal fibroblast cell (L929) [21]. After cell adhesion (1 × 10^4^ cells/well) for 24 h in 96-well plates, extracts were added (0.5, 1, 2.5, 5, 7.5, 10, and 20 µM) and incubated for 24 h and 48 h. By spectrophotometry (490 nm, Molecular Devices, CA, USA), optical density was evaluated, and cell viability was determined with respect to the control (100%) [22].

#### 2.6.3. Hydrogen Peroxide-Induced Oxidative Stress in L929 Fibroblast Cells

At a density of 1 × 10^4^ cells/well, L929 fibroblast cells were seeded with DMEM medium, supplemented with 10% FBS, and incubated with 5% CO_2_ at 37 °C overnight. Then, 0.5, 1, 2.5, 5, 7.5, 10, and 20 µM of extracts rich in anthocyanins were added to these wells concomitantly with 1.0 mM H_2_O_2_ to treat exposed cells, and cells incubated for 24 h [23]. Cell viability was evaluated using an MTT assay as described in Leonarski et al. [5].

#### 2.6.4. Antitumoral Activity

Using an MTS colorimetric assay, cell viability was measured. In 96-well plates, mouse glioma (GL261) and lung adenocarcinoma (A549) cells were seeded at a density of 1 × 10^4^ cells/well and placed in a CO_2_ incubator for 24 h at 37 °C for attachment. After 24 h, the cells were treated with 2.5 µM of extracts and doxorubicin (5 µM) as a positive control. Then, cells were incubated at 37 °C in CO_2_ for 24 h and 48 h. Then, the medium was removed, and the cells were washed twice with PBS. In total, 100 μL of medium and 20 μL of MTS reagent were added and incubated for 2 h at 37 °C with 5% CO_2_. The cells’ optical density (OD) was measured on plates at 490 nm [24].

### 2.7. Statistical Analysis

The dataset was evaluated by a one-way analysis of variance (ANOVA). Using *t*-test or Tukey test, significant differences were determined at a probability level of less than 5% (*p* < 0.05). All statistical procedures were performed using Statistica v. 13.5 software (TIBCO Software Inc., Palo Alto, CA, USA). The results were presented as the mean ± standard deviation.

## 3. Results and Discussion

### 3.1. Laboratory Scale-up

The use of new technologies for food processing, such as UAE, aims to save energy, reduce time, and improve product quality and shelf life [15]. In studies by Carail et al. [15] and Gille et al. [25], the authors verified the use of UAE for carotene extraction, using Equations (1) and (2) to calculate the ultrasonic intensity (UI) to verify its effect on possible carotene degradation. In our study, these equations were used with the main objective of scaling up the process.

For scale-up (using the 300 mL reactor), the use of 450 W achieves a maximum ultrasound intensity (UI) equal that of 300 W for the 15 mL reactor (Table 1). Therefore, for a 15 mL vessel, the extraction of anthocyanins from black rice bran at 50 °C, using a 60:40 ratio of 0.1 mol/L citric acid–ethanol and a power of 300 W, obtained 1.97 mg C3G/g DW of anthocyanins (anthocyanin recovery (AR) = 67.7%). For the laboratory scale-up, the same conditions of time, temperature, solvent, and a power of 450 W were used for volumes of 150 mL (10× increase) and 300 mL (20× increase), obtaining 1.94 mg C3G/g DW (AR = 66.7%) and 1.82 mg C3G/g DW (AR = 62.7%) of anthocyanins, respectively.

In a study by Belwal et al. [11], in which anthocyanins were extracted from *Pyrus communis* ‘Starkrimson’ fruit peel, the authors scaled up from 30 to 150 mL (5× increase), 450 mL (15× increase), and 3000 mL (100× increase). The authors obtained comparisons with the 30 mL reactor, where were around 92% for the 150 mL reactor, 88% for the 450 mL reactor, and 83% when increasing the reactor to 3 L. In our study, compared with the extraction in the 15 mL reactor, there was no significant difference for extractions in a reactor that had a 150- or 300-mL volume, which recovered around 98 and 92.5% of anthocyanins, respectively.

To increase the scale, whether to a pilot or industrial scale, we needed to use equipment that has a greater variation in power. In the study by Chen et al. [26], the authors carried out an increase in scale for anthocyanins extracted from purple corn bran, from 50 mL to 42 L, using ultrasound, obtaining a small decrease of 5.46% in the anthocyanins extracted compared to the laboratory scale. According to the authors, the results obtained suggest that scaling up to an industrial scale of ultrasound extraction is easily achieved.

### 3.2. Partial Purification of Anthocyanins Extracted from Black Rice Bran

Amberlite XAD-7HP stands out in terms of separating anthocyanins of all the resins available for partial purification. In studies by Das et al. [13], Heinonen et al. [27], and Chen et al. [12], of the several resins used for purifying anthocyanins from different sources (purple rice bran, purple-fleshed potato, and mulberry, respectively), the one that stood out was Amberlite XAD-7HP, which presented better adsorption and desorption characteristics compared to other resins. The efficient elution of the solute adsorbed to the resin is important to guarantee the use of the resin over multiple cycles [28].

For the partial purification of the extract, two organic acids (acetic and citric acid) were used, in a 1% volume, to desorb the anthocyanin. According to Chang et al. [29], citric acid solution, as an eluent, is preferred to hydrochloric acid, as it avoids traces of this acid in the product after purification. Among the acids used as an eluent, acetic acid showed better results, recovering about 57.2% of the total anthocyanins after purification. In contrast, with citric acid that recovery is 36.8% of the total anthocyanins. Adding acid during anthocyanin elution is necessary for these compounds to maintain a stable form (flavylium cations) and prevent degradation [30]. In their flavylium cation form, anthocyanins have a lower affinity for the resin; therefore, the addition of acetic acid possibly led to a greater formation of flavylium cations, which resulted in a higher purification yield.

In a study by Contreras et al. [31], a raw extract obtained from native black beans after purification with Amberlite XAD-7HP, using acidified ethanol/water 70/30 *v*/*v* (0.3% formic acid) as eluent, reached a 53.8% recovery of anthocyanins, a result close to that reported in our study using acetic acid (1%). In the study by Das et al. [13], the authors reported an efficiency in the recovery of anthocyanins extracted from purple rice bran of 41.5%, using 95% ethanol for adsorption; these values are closer to those reported when using citric acid.

In a study by Zhao et al. [32], the authors observed that most anthocyanins from black peanut skin were eluted from the column early due to their high polarity and low adsorption capacity to Amberlite XAD-7HP resin. The use of acidified ethanol (40–75%, *v*/*v*) to recover anthocyanins in the purification of Amberlite XAD-7HP can be considered an efficient strategy [12,27,32].

### 3.3. Comparison of Crude and Partially Purified Anthocyanin Extracts

The crude anthocyanin extract achieved 1.06 mg/g of TMA, while the partially purified extract increased the anthocyanin content by 4.2-fold (4.44 mg/g) (Table 2). The same behavior was observed for cyanidin-3-glucoside, for which the crude extract presented 1.03 mg/g (about 98% of the total anthocyanins) and purified extract presented 3.97 mg/g (corresponding to 89.5% of total anthocyanins).

In the study by Das et al. [13], XAD resin was used to purify purple rice bran extracts, verifying a 5.5-fold increase for the purified extract, while, for C3G, the increase was 6.6-fold. In a study by Jeyaraj et al. [33], in which the Amberlite resins XAD-16 and C18-OPN were used to purify an extract of *Clitoria ternatea* flower, the concentration of anthocyanins was found to be approximately 4.5–4.7 times higher; values close to those reported in our study.

It was also observed that the purification process improved in vitro antioxidant activity (DPPH and ABTS). It can be seen in Table 2 that the IC_50_ for the crude extract was 1.57 and 1.26 mg/mL for the DPPH and ABTS radicals, respectively. These values decreased to 0.67 and 0.33 mg/mL for the DPPH and ABTS radicals in the partially purified extract, respectively. This increase in antioxidant activity is associated with the C3G concentration discussed previously.

In a study by Chumchoochart and Sutthanut [34], the authors reported an IC_50_ value for DPPH equal to 1.69 mg/mL, and in a study by Singha et al. [35], the authors reported values between 0.10 and 1.25 mg/mL; both corroborate those found in our study. Sansenya and Nanok [36] reported purified fractions of black rice IC_50_ values between 0.35 and 0.51 mg/mL for the ABTS radical, a value close to that of the purified sample presented in our study.

### 3.4. Effects of Crude and Partially Purified Extracts on In Vitro α-Amylase and α-Glucosidase Inhibition 

**Figure 2:**
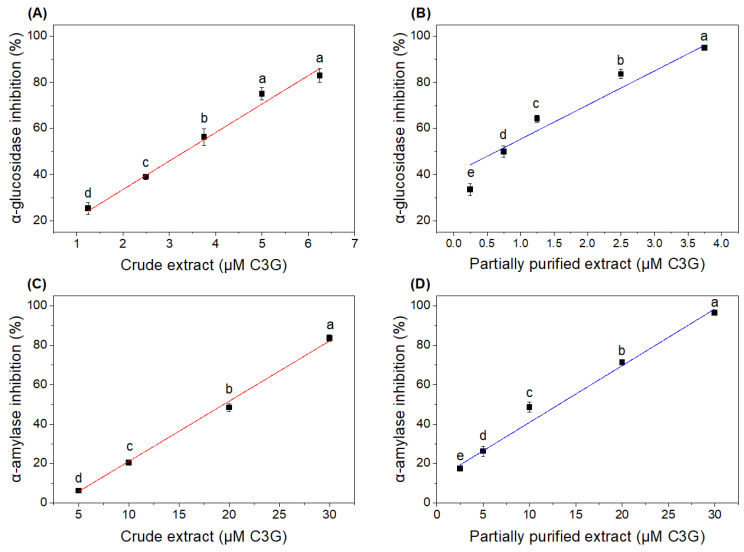
Inhibitory effects of (**A**) crude and (**B**) partially purified anthocyanin-rich extracts on α-glucosidase, and of (**C**) crude and (**D**) purified anthocyanin-rich extracts on α-amylase. Different letters indicate significant differences by Tukey test (*p* < 0.05).

Figure 2 shows the black rice bran extracts’ in vitro enzymatic inhibition values for α-glucosidase and α-amylase. Partial purification improved significantly (*p* < 0.05) the inhibitory effect of the extract. For the inhibition of α-glucosidase, an IC_50_ value of 3.23 µM (1.45 mg/mL) was obtained for the crude extract and 0.82 µM (0.37 mg/mL) for the partially purified extract, improving its activity by approximately 4-fold after purification. Shimoda et al. [37] reported an IC_50_ value for α-glucosidase of 0.41 mg/mL for a purple rice extract, while Choi et al. [38] presented, for C3G (the main compound from black rice extract), an IC_50_ of 13.7 µM for α-glucosidase.

For α-amylase inhibition (Figure 2C,D), the IC_50_ was found to be 19.31 µM (8.67 mg/mL) for the crude extract, while for the partially purified extract it was 12.5 µM (5.60 mg/mL), meaning they differed significantly from each other (*p* < 0.05). Values close to the IC_50_ for α-amylase inhibition were reported by Aalim et al. [39] for black rice grains (8.36 mg/mL). 

According to Choi et al. [38], the intestinal enzymes α-glucosidase and pancreatic α-amylase are responsible for the hydrolysis of various carbohydrates (starch, glycogen, sucrose, etc.), and the inhibition of these enzymes leads to a delay in the rise of blood glucose levels. The degradation of this starch would lead to high postprandial hyperglycemia [40]. This delay in hydrolysis through enzyme inhibition is a therapeutic approach to controlling postprandial hyperglycemia in pre-diabetes, diabetes, and obesity [41,42].

The inhibition of α-amylase or α-glucosidase enzymes occurs because anthocyanins enter their active site and reduce their catalytic action through hydrogen bonding [43]. In a study by Sui et al. [7], it was found that C3G made seven hydrogen bonds with porcine pancreatic α-amylase and had the highest inhibition activity among the four anthocyanins studied (cyanidin-3-glucoside, cyanidin-3,5-glucoside, cyanidin-3-rutinoside, and peonidin-3-glucoside).

Drugs used to treat diabetes can have undesirable effects, such as causing hypoglycemia in higher doses, liver problems, lactic acidosis, and diarrhea [42]. Therefore, many studies have suggested the use of natural compounds that can replace conventional medicines and consequently lead to a reduction in side effects [38,42].

### 3.5. Cell Culture

#### 3.5.1. Cytotoxicity

**Figure 3:**
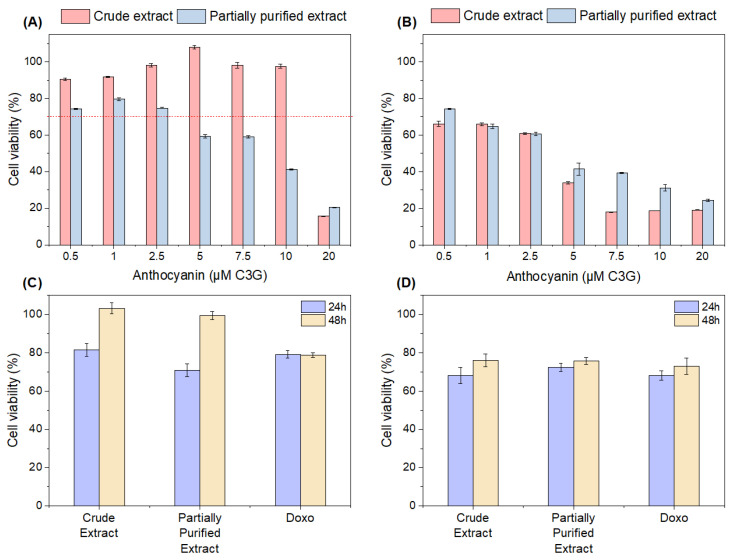
(**A**) Cytotoxicity of L929 cells treated with the crude and partially purified extracts. The dotted line corresponds to 70% cell viability (below 70%, the extract is considered cytotoxic); (**B**) viability of L929 cells treated with the crude and partially purified extracts with concomitant H_2_O_2_ exposure; (**C**) antitumoral activity (%) of crude and partially purified extracts in mouse glioma (GL261) cells, and (**D**) in lung adenocarcinoma (A549) cells.

A cytotoxicity assay was performed using L929 cells; the results are presented in Figure 3A. For crude extracts, only 20 µM presented a cell viability lower than 70%, while, for the partially purified extracts, volumes higher than 2.5 µM presented a cell viability lower than 70%. According to ISO 10993-5:2009 [21], an L929 cell viability higher than 70% does not show cytotoxicity. The semi-purified extract may have concentrations of other compounds that were not removed in previous steps, which could have affected its cytotoxicity, such as the acetic acid used to elute the extract, or even traces of other solvents. However, further studies must be carried out to identify these compounds.

Sangkitikomol et al. [44] verified the cytotoxicity of a black rice extract in HepG2 cells at concentrations above 800 µg/mL. In Aprodu et al.’s [45] study, at concentrations greater than 800 µg/mL of microencapsulated black rice extracts, a significant decrease was observed but without cytotoxicity (a cell viability of L929 cells of around 80%). In a study by Wang et al. [46], it was verified using L929 cells that the anthocyanins from their blueberry extract showed cytotoxicity (viability less than 70%) at a concentration of 800 µg/mL. 

Toxicity analysis is important to verify the concentration at which extracts may be harmful in their application. Alongside this, oxidative stress analysis and antitumor activity were carried out to verify the biological potential of these extracts.

#### 3.5.2. Effects of Crude and Partially Purified Extracts on Cell Viability in H_2_O_2_-Induced L929 Cells

Oxidative stress was achieved in L929 cells using hydrogen peroxide (H_2_O_2_). At the concentration of 1 mM H_2_O_2_, cell viability was approximately 11.2%. By adding the crude and partially purified extracts, the cytoprotection of the L929 cells was verified (Figure 3B). At low concentrations (0.5, 1, and 2.5 µM), around a 60–74% cytoprotection of the L929 cells was observed, and this was higher for the partially purified sample at a concentration of 0.5 µM, significantly differentiating it from the crude extract (*p* < 0.05). With increasing concentrations, there was a decrease in the cytoprotection of both samples, which was more pronounced for the crude extract. In this case, it was shown that small concentrations of the extracts are efficient at carrying out cell cytoprotection. 

Ereminas et al. [47] reported a cytoprotective effect of C3G on rat C6 glial cells at concentrations of 5, 10, and 20 µM, ranging from 58 to 65% (H_2_O_2_ 100 µM) and not differing significantly from each other. In a study by Zhang et al. [48], C3G from Chinese Bayberry showed a cytoprotection of around 70% in β cells (INS-1) (with 1 mM H_2_O_2_) at concentrations of 0.5 and 1 µM, which decreased significantly (*p* < 0.05) to around 60% when the concentration was increased to 5 µM. According to Tan et al. [49], the cytoprotective effect of C3G is death receptor-dependent, as is obtained by regulating two apoptotic pathways: the mitochondrial pathways and the external pathway.

For anthocyanin-rich extracts from black rice bran obtained by pressurized liquid extraction (PLE) and heat-stirring extraction (HSE), a cytoprotection of around 80% was also evidenced in L929 cells when using 250 µg/mL [5]. Palungwachira et al. [50] showed an improvement (around 25% for 10 µg/mL of extract and 10% for 25 µg/mL) in the cytoprotection of rat dermal fibroblast (RDF) cells (with 0.6 mM H_2_O_2_) by black rice extracts purified with a C18 Sep-Pak cartridge compared to the crude extract.

Due to their antioxidant potential, their cytoprotective effect comes from the anthocyanins present in the extracts [5]. This activity is related to the capture of the free radicals (H_2_O_2_) responsible for tissue damage; therefore, the extract has the potential for application in wound healing [23,51]. However, more studies are needed to explore this potential in full.

#### 3.5.3. Antitumoral Activity

Antitumoral activity (or antiproliferative activity) assays were performed using mouse glioma (GL261) cells and lung adenocarcinoma (A549) cells (Figure 3C,D). A concentration of 2.5 µM was used for both extracts (a concentration that did not show cytotoxicity according to Section 3.5.1), and 5 µM doxorubicin (Doxo) was used as the standard antibiotic.

In terms of antitumoral activity in GL261 cells, within 24 h the samples showed a decrease in metabolic activity of around 20–30%, a value similar to the antibiotic Doxo, and did not differ from each other. However, within 48 h, the cells began to increase again, recovering to their initial state (close to 100%) for both extracts, while, for Doxo, the activity remained at around 80%. Therefore, although both extracts showed initial promise, over time the concentration was not sufficient to cause a significant effect on the metabolic activity of GL261 cells.

For A549 cells, it was found that both extracts presented effects between approximately 68 and 76%, while the antibiotic Doxo presented similar values, between 68% and 73%, with no sample differing from the others. Although the result was not as expressive, similar results were obtained for the antibiotic. In a study by Xue et al. [52], when evaluating the antitumor activity in A459 cells of raspberry wine residue extract at a concentration of 2.5 µM, the authors showed a decrease in cell viability of between approximately 15% and 28% within 48 h, results close to those reported in our study. Therefore, our extracts have the potential for application against A549 cells. 

Further studies have shown that C3G (the main compound of black rice bran extracts) inhibited the proliferation, migration, and invasion and promoted the apoptosis of A549 cells, processes that involve cancer metastasis [53,54]. The negative regulation of TP53I3 and the inhibition of the PI3K/AKT/mTOR pathway carried out by the anthocyanin C3G are responsible for inhibiting the proliferation, migration, and invasion and also facilitating the apoptosis of A459 cells [53]. Although the extracts have potential in treating lung adenocarcinoma, more studies are needed to highlight their possible mechanisms and effectiveness.

## 4. Conclusions

It was possible to increase the laboratory scale for ultrasound-assisted extraction (UAE) by 20 times without significantly affecting the recovery of anthocyanins from black rice bran (around 63% were recovered). Furthermore, their partial purification using a microporous resin (Amberlite XAD-7HP) resulted in an extract with a higher total level of anthocyanins (by 4.2-fold), and greater antioxidant power (DPPH and ABTS). In addition, the partially purified anthocyanin extracts presented an antidiabetic effect greater than that of the crude extract (being approximately 4 times higher for α-glucosidase and 0.65 for α-amylase). No cytotoxicity was found towards L929 cells for the crude (concentration ≤ 10 µM) or partially purified extracts (concentration ≤ 2.5 µM). For oxidative stress, low concentrations of both extracts (0.5, 1, and 2.5 µM) were sufficient to protect about 70% of L929 cells. Around a 25–30% antitumor activity was found in lung adenocarcinoma (A549) cells for both extracts. Therefore, further studies on the extract should be conducted to verify whether this compound can be used as an alternative therapeutic approach to control hyperglycemia in diabetic patients or as a potential anti-cancer supplement.

## Figures and Tables

**Table 1:** Instrumental parameters for laboratory scale-up.

Frequency Power (W)	Real Frequency Power (W)		Ultrasound Intensity (W/cm^2^)	
	15 mL Reactor	300 mL Reactor	15 mL Reactor	300 mL Reactor
300	24.61	71.31	3.48	2.52
350	27.89	78.04	3.95	2.76
380	28.85	80.58	4.08	2.85
400	30.99	85.69	4.39	3.03
450	32.18	88.31	4.55	3.12

**Table 2:** Analysis of the anthocyanin-rich extract of black rice bran before and after purification.

Analysis	Extracts	
	Crude	Partially Purified
TMA (mg/g)	1.06 ± 0.04 ^b^	4.44 ± 0.60 ^a^
C3G (mg/g)	1.03 ± 0.01 ^b^	3.97 ± 0.02 ^a^
DPPH IC_50_ (mg/mL)	1.57 ± 0.03 ^a^	0.67 ± 0.01 ^b^
ABTS IC_50_ (mg/mL)	1.26 ± 0.01 ^a^	0.33 ± 0.01 ^b^

Results show the mean ± standard deviation. TMA: total monomeric anthocyanins; C3G: cyanidin-3-glucoside. Different letters indicate significant differences by *t*-Test (*p* < 0.05).

## Data Availability

The data are contained within this article.
